# Contesting crisis narratives amidst climatic breakdown: Climate change, mobility, and state-centric approaches to migration

**DOI:** 10.3389/fsoc.2024.1411683

**Published:** 2025-01-09

**Authors:** Kenna Sim-Sarka

**Affiliations:** Research Institute for Migration, Ethnicity and Society (REMESO), Institute for Culture and Society, Linköping University, Norrköping, Sweden

**Keywords:** climate change, climate migration, climate mobilities, methodological nationalsim, migration studies

## Abstract

Human mobility in the context of climate change is often identified as one of the largest future impacts of the climate crisis. It is often assumed by international institutions and national governments that climate change will drive mass migration movements across borders, leading to a prioritization of research that aims to predict future climate migration to aid border security and the creation of migration policies. This article focuses on knowledge production research concerning around climate-related mobility and how knowledge being produced upholds state-centric approaches to migration and migration management. It argues that by leaving state-centric approaches to migration unquestioned in the name of managing climate-related mobility, national governments and other institutions reproduce inequalities for those who are in the nexus of migration and climate change. This article considers alternative conceptions of mobility and climate change, including the climate mobilities paradigm and decolonial understandings of migration, and how these can shift our analytical focus to more holistic and decolonial understandings of migration.

## 1 Introduction

Human mobility in the context of climate change, including migration, displacement, and relocation, is often identified as one of the largest future impacts of the climate crisis. In light of continued record-breaking temperatures, increased natural disasters, and the absence of robust action on reducing global emissions, there is profound concern over the realities of a world in which the global temperature may well increase by 1.5°C or more by 2050. Climate-related mobility is a burgeoning area of research, policy, and knowledge production, bringing together scholars from a variety of disciplines in both environmental and social sciences, policy actors in the field of human mobility, as well as global climate change politics (Nash, 2018). In this sense, climate change and mobility are increasingly being constructed as an interconnected web of global risks (Bettini, 2013b; Nash, 2018).

It is often assumed by dominant climate migration narratives that climate change will drive mass migration movements across international borders, representing a major geopolitical risk and leading to conflicts over resources (Mayer, 2013; Baldwin et al., 2014; Bettini, 2014; Boas et al., 2019). Therefore, intergovernmental organizations, national governments, and large-scale institutions, overwhelmingly located in the Global North, seek to understand climate migration in the interests of border security and the creation of new migration policies that react to these phenomena. This has led to a prioritizing of climate migration research that aims to predict future climate migration flows and sites likely to be a ‘source’ of climate migrants (Nash, 2018; Boas et al., 2019). At the same time, such conceptions have been long critiqued by critical climate migration scholars for a variety of reasons, including understanding climate migration through an environmentally determinist lens (Gemenne, 2011; Nicholson, 2014), presenting migration and future climate migrants as a crisis that needs to be managed and governed (Bettini, 2014; Durand-Delacre et al., 2021), and failing to consider to the root causes of climate-related risk, namely capitalism, colonial, and unequal power relations between the Global North and the Global South (Baldwin, 2022). Arising out of these critiques, recent research has focused on new conceptual frameworks of human mobility in light of climate change, calling on researchers and policymakers to de-exceptionalize climate migration, including the climate mobilities perspective and decolonial perspectives of migration and climate change (Baldwin et al., 2019; Whyte et al., 2019; Boas et al., 2022).

Despite these long-standing critiques and new perspectives being forged, multilateral institutions and national governments, many of which are located in the Global North, remain focused on presenting climate migration as a looming crisis that needs to be managed and controlled, therefore calling on more research to aid in the creation of policy. The United States Department of Defense (2015) has identified migration and displacement, both within and across state borders, as responsible for negative human security outcomes. A report by the Noonan and Rusu (2022) stated that mass migration due to climate change represents a major geopolitical risk, and therefore a priority of the EU is prevention of future large-scale climate migration. Instead of challenging these assumptions, public funding schemes for scientific research intended to inform policy development continue to perpetuate this emphasis on securitization and managing migration (Boas et al., 2019).

In migration studies, scholars have long critiqued both the epistemological and ethical problems which flow from an understanding of migration that frames migration as a problem (Sheller and Urry, 2006; Dahinden, 2016; Anderson, 2019). Such perspectives stem from critiques of methodological nationalism, defined by Wimmer and Glick Schiller (2002) as an epistemic bias in which the nation state is assumed to be the natural container for social order. Such lines of inquiry have interrogated the naturalization of the nation-state and citizen/migrant binaries. While some scholars have brought critiques of methodological nationalism present in migration studies in to conversation with research on climate-related mobility (see Sheller, 2018; Boas et al., 2024), this area remains underexplored.

With this background in mind, this article centers on how critical perspectives in migration studies, rooted in both critiques of methodological nationalism and decolonial perspectives of migration and migration studies, can enrich research on climate-related mobility. It seeks to identify how knowledge produced about climate change and mobility upholds state-centric approaches to migration and migration management. By state-centric views of migration, I refer to conceptions of migration and mobility that naturalize the nation-state as the given referent for social organization and uncritically frame migration as a problem for national governments to manage, rooted in methodological nationalism (Scheel and Tazzioli, 2022). I argue that by leaving these state-centric approaches uninterrogated in the name of managing climate-related mobility, national governments and other institutions reproduce inequalities for those who are in the nexus of migration and climate change (Bates-Eamer, 2019; Stanley, 2021). Further, in this article I aim to bring both the climate mobilities approach (Boas et al., 2022) and decolonial perspectives of migration and climate change into conversation with critical perspectives in migration studies. My intention is not to forge a new theoretical perspective, but rather to contribute to climate mobilities research by bringing these lines of inquiry and theoretical dimensions from different, yet related, fields together to highlight how critiques of methodological nationalism and decolonial perspectives in migration studies can enrich climate change and mobility research.

While an extensive review of these histories and contemporary manifestations is outside the scope of this article, this article is situated in an understanding of climate change, climate change adaptation, and climate-related migration as outcomes of coloniality, global capitalism, and global hierarchies between the Global North and Global South (Davis and Todd, 2017; Baldwin, 2022; Perry and Sealey-Huggins, 2023). Historical perspectives have highlighted how subjugation and exploitation of the Earth was entwined with the creation of a racial hierarchy based on Indigenous dispossession and the enslavement of Black people, as well as how industrialization and the rise of the coal industry was dependent on the plantation economies and imperial plunder being waged in the colonies (see Yusoff, 2018; Ferdinand, 2022). Resultingly, the global capitalist order systematically abuses nature and exploits large segments of the population, including racialized communities (Pulido, 2017; Gonzalez, 2020). As argued by Nixon (2013) in his concept of slow violence, the outcomes of climate change and environmental degradation unfold gradually and disproportionately affect those in the Global South and racialized communities. Further, climate change solutions prioritize green capitalist expansion on terms that satisfy transnational financial and political interests, resulting in communities that are most affected by climate change then being subject to solutions that further continue to extract value (Paprocki, 2018; Morris, 2019; Perry and Sealey-Huggins, 2023; Anantharajah, 2024).

The article will be structured as follows. First, I will overview what is meant by state-centered understandings of migration and new approaches in migration studies which critique methodological nationalism, including the new mobilities paradigm, methodological de-nationalism, and decolonial perspectives of migration. Next, I will provide a background of the genealogy of climate migration as a concept and an object of research, illustrating how climate migration research has often held state-centric views towards migration. I will then overview two different approaches to climate change and mobility, the climate mobilities paradigm and decolonial understandings of climate change and migration, bringing other perspectives from migration studies into dialogue with these approaches. I will then initiate a discussion on how contemporary forms of migration management expose those in the climate-mobility nexus to precarity and insecurity, amounting to climate injustice and institutional violence, highlighting what is at stake in these debates.

## 2 Critiques of methodological nationalism in migration studies and new approaches

Scholars in migration studies have long critiqued the centrality of the nation-state in migration research and have developed new approaches in response to these critiques. Numerous scholars have noted that the now hegemonic nation-state stance on migration arose out of historical, social, and political processes (Wimmer and Glick Schiller, 2002; Czajka, 2014; Dahinden, 2016; Scheel and Tazzioli, 2022). In charting the genealogy of nation-states, attention has been called to how the birth of the modern nation-state order does not immediately arise from the 1,648 Treaty of Westphalia as thought in traditional accounts, but rather the emergence of the modern nation-state system and international order is much more recent and entwined with empire (Czajka, 2014; Hansen, 2022). The consolidation of the ‘national order of things’ (Malkki, 1995) became hegemonic after the First World War in which processes of nation building fostered a new conception of ‘the people.’ As a consequence, distinctions between the ‘citizen’ and the ‘migrant’ become key to the creation and governing of nation-states, and migrants began to be perceived as essentially different subjects who continued to hold memberships of their ancestral homelands, or be considered as foreigners (Anderson, 2019; Scheel and Tazzioli, 2022).

Wimmer and Glick Schiller (2002) have defined methodological nationalism as an epistemic bias in which the nation state is assumed to be the natural container for social order. Methodological nationalism can manifest in several ways in social science research, including failure to consider how nationalism and the creation of nation-states has shaped social and political life, naturalizing the nation-state as the universal mode of political organization, and limiting social scientific analysis to the territorial boundaries of the nation state. It has been long noted how the biases of methodological nationalism and state-centered perspectives of migration have seeped their way into migration studies, including an uncritical acceptance of state-centered understandings of migration (Dahinden, 2016; Anderson, 2019; Scheel and Tazzioli, 2022).

Scheel and Tazzioli (2022, p. 6) define state-centered understandings of migration as ‘conceptions of migration that make the division of the world into a set of mutually exclusive nation-states the unquestioned reference point for the determination of what migration is.’ Bounded up in this is the pervasive ignorance of how the formation of modern nation-states has influenced predominant understandings of migration. In state-centered understandings of migration, it is assumed and accepted that it is the sovereign right of states to control and determine access into their territory for the sake of national security, protection of the labour market and national community (Friðriksdóttir, 2017; Oelgemoller, 2017). Embedded in this is the naturalization of distinctions between ‘citizens’ and ‘migrants’ and the presumption that migrants have a capacity to disrupt the nationally bounded receiving society which is viewed as culturally homogenous (Dahinden, 2016; Anderson, 2019). Such assumptions reify the framing of migration as a problem for national governments and as a security issue in need of monitoring, regulation, and control. Further, the nation-state view on migration perpetuates the idea that migrants need to be integrated in the nationally bounded receiving society. As summarized by Scheel and Tazzioli (2022), statist conceptions of migration thus invisibilize nation-state practices of bordering and boundary-making that enact some people as migrants in the first place.

Such perspectives have also critiqued contemporary practices of migration management in which nation-states, particularly liberal democracies located in the Global North, have increasingly shifted to migration management practices centered around deterrence of migrants deemed ‘undesirable’, increased temporariness, and the restriction of inclusion into the nation-state (Oelgemöller, 2011; De Haas et al., 2018; Cook-Martín, 2019; Triandafyllidou, 2022). Such measures include externalizing and deterritorializing borders (Tazreiter, 2015; Casas-Cortes et al., 2016; Moreno-Lax and Lemberg-Pedersen, 2019), embracing circular labor migration and temporary visa schemes (Tazreiter, 2019; Akbar, 2022), limiting access to state services for those with temporary status (Koleth, 2017; Lafleur and Mescoli, 2018; Rogat, 2022; Näre and Maury, 2024), and the erosion of permanent residence for asylum seekers (Schultz, 2020; Stoyanova, 2022). Such practices are often traced to mechanisms of borders and bordering, rooted in ‘technologies of control within broader logics of governmentality and management’ (De Genova et al., 2014, p. 57). Critical migration scholars have also made explicit how such mechanisms of bordering function as a multiplication of labor (Mezzadra and Neilson, 2013) and disciplining and securing labor flows and people in light of colonial and racial divides (Georgi, 2019; Schinkel and Van Reekum, 2024), suppressing wages and creating a continuous supply of easily exploitable labour.

Arising out of these critiques, scholars have developed theoretical orientations that aim to both de-naturalize the nation-state and de-exceptionalize migration. The development of the new mobilities paradigm, developed by John Urry (2010) and others (Sheller and Urry, 2006; Faist, 2013), sought to challenge the fixedness of territories and the view that sedentarist lifestyles are the norm. The ‘mobilities turn’ aimed to develop ‘a sociology which focuses on movement, mobility, and contingent ordering, rather than upon stasis, structure, and social order’ (Urry, 2007, p. 18). Hoffmeyer-Zlotnik (2024, p. 1304) argues migration and mobility have different conceptualizations of human movement, with migration centering the perspective of place and mobility centering the perspective of flow; from the mobility or flow perspective, ‘movement happens in the context of a general norm of mobility, not only of humans but of ideas, goods, and capital, among others.’ Such work has linked different forms and scales of movement, as well as paid attention to the ongoing yet temporary mobile formations that shape social life (Sheller, 2018). The articulation of regimes of mobility also highlights how certain (im)mobilities are privileged or stigmatized and how these are entangled in and shaped by different power systems (Glick Schiller and Salazar, 2013).

Migration scholar Anderson (2019, p. 2) calls attention to the ‘ethical and epistemological challenge posed by methodological nationalism’ that confounds migration research; that is, the consistent framing of migration and migrants as a problem for national governments and host societies and taken-for-granted distinctions between citizens and migrants. However, Anderson recognizes that simply shedding the categories of migrant and citizen should not be the response to realizing the constructed nature of these categories, as such distinctions matter both normatively and empirically. She develops the approach of ‘methodological de-nationalism’ which ‘makes visible and investigates the workings of state-imposed categories of migrant and citizen in all their differentiations, their impacts on the experiences of individuals and groups, and the management, governance and accountability of nationalized territories and international/global relations more generally’ (Anderson, 2019, p. 6). One strategy for methodological de-nationalism is what Anderson calls ‘migrantizing the citizen,’ or paying attention to the ways in which the relations between immigration, race, nationality and class reinforce and contest each other, and how this in practice complicates the migrant/citizen binary. To illustrate this, Anderson gives the example of governmental controls over the mobility of poor and racialized populations who are also citizens; in a methodological de-nationalism framework, migration control can be seen as one of the various ways in which people’s movement has been guided and constrained over centuries.

Decolonial approaches to migration studies have also critiqued the centrality of state-centric understandings of migration and migrant categories. As explained by Maldonado-Torres (2011), decolonial thought is not part of one theoretical school, but rather branches out into a family of diverse positions which understand coloniality as a fundamental problem of the modern age. Embedded in this is an understanding of coloniality as a matrix of power that uses race as an instrument of social classification and control of labour, as well as the importance of knowledge production and the subjugation of knowledge production in maintaining colonial hierarchies (Quijano, 2007; Mignolo, 2011). Decolonial perspectives in migration studies have been instrumental in analyzing and articulating how migration trajectories are shaped by histories and ongoing manifestations of colonialism (Coleborne, 2015; Gutiérrez Rodríguez, 2018; Achiume, 2019), challenging ‘universal’ concepts, theories, and methods in migration studies and how these can be northern-centric and marginalize other understandings of migration and movement (Ramirez, 2020; Itzigsohn, 2023; Vanyoro, 2024), and critiquing migration studies methodologies (James, 2016; Raghuram and Sondhi, 2023). Decolonial perspectives have emphasized how a study of migration that begins at the inception of the nation-state erases histories and epistemologies of movement that supersede the development of the nation-state (Whyte et al., 2019; Ramirez, 2020), as well as how concepts such as migrant, citizen, borders, and migration itself have largely been developed through a Northern perspective.

Approaches to understanding migration in ways that both de-naturalize the nation-state and national borders and de-exceptionalize migration are particularly relevant for issues surrounding climate change. As noted by Boas et al. (2024), the effects of climate change are not bound to and posing new challenges for anthropogenic borders; as such, the era of climate change is tied in with conceptual questions about territoriality, borders, and mobilities.

## 3 Knowing climate change and migration

It has repeatedly been demonstrated that human activities, particularly those located in industrial economies based on fossil fuel extraction and consumption, have irrevocably changed the climate. According to the Intergovernmental Panel on Climate Change, climate change is already having an impact on weather and climate extremes across the globe, including increased heat waves, heavy precipitation, droughts, and tropical cyclones (IPCC, 2022). While there is significant research on the present and potential impacts of climate change, it is still debated by researchers how climate change affects migration decisions and the movement of people in a changing world. Humans have moved in response to changes in the environment since time immemorial, and in this sense climate migration is not an exceptional phenomenon but one adaptive strategy of many (Baldwin et al., 2019; Whyte et al., 2019). However, ‘climate migration’ as a concept has become a subject of great interest to researchers, policy makers, politicians, journalists, and the public to understand how climate changes will impact human mobility. Increasingly, researchers have critically examined how knowledge about climate change and migration is produced and what kinds of knowledges are being uplifted, particularly in regard to how climate migration research can reproduce already existing relations of inequality.

Climate change and migration has a lengthy prehistory, and the nexus between ecological change and human mobility has been a topic of analysis since the 1800s; however, interest in the connections between environment and mobility waned in the first half of the twentieth century and then picked up again in the latter half (Piguet, 2013). Recent accounts for the genealogy of ‘climate migration’ note that it was not the case that migration researchers rediscovered environmental considerations, but rather international actors drove this renewed interest (Baldwin, 2022). Increased consideration of the interplay between ecological conditions and human mobility started to develop in the 1970s, as NGOs and political thinktanks began considering the impacts environmental degradation could have on humans in the long term, coining the term ‘environmental refugee’ (Boano et al., 2008; Bettini, 2013b). Such developments also coincided with heightened concern and anxieties over increasing numbers of migrants and refugees and destabilization to the global national order brought about by global decolonization movements (Malkki, 1995; Oelgemoller, 2017; Baldwin, 2022). During the 1990s, as climate change garnered more attention as a future challenge to ecosystems and human livelihoods, displacement and migration due to climate change expanded in significance amongst governments, academics, NGOs, and environmental activists. IPCC (1992, p. 103) predicted that the ‘the gravest effects of climate change may be those on human migration as millions are displaced by shoreline erosion, coastal flooding, and severe drought.’

Such statements led to increased research attempting to estimate the severity and impacts of climate migration and quantitively predict the number of future climate migrants, including Norman Myers (1997, 2002) who forecasted there would be 200 million climate refugees by 2050. A number of influential reports from the fields of international development detailed how in the coming decades there would be a mass migration of ‘climate refugees,’ displaced due to rising sea levels, droughts, and natural disasters (Jacobsen, 1988; Christian Aid, 2007; Environmental Justice Foundation, 2009). Such descriptions of looming mass migration were linked to national sovereignty and human security, as it was argued that large numbers of climate refugees would exacerbate global conflicts and put a strain on host countries’ resources (Homer-Dixon, 1991). Further, the narrative of mass climate-induced migration was often invoked by NGOs, activists, and others to illustrate a ‘human face’ to the implications of climate change, often in an attempt to convince governments to take climate change seriously (Baldwin et al., 2014; Ransan-Cooper et al., 2015). Such perspectives held an environmentally deterministic approach, in which it was assumed that climate change was *the* cause of migration in environmentally vulnerable areas (Gemenne, 2011; Bettini, 2013b). Additionally, many researchers assumed that climate migration would usually take the form of cross-border migration from South to North. As summarized by Bates-Eamer (2019, p. 198), ‘The result was an oversimplified and highly politicized understanding of new dynamics driving an old phenomenon.’

Researchers in migration studies, human geography, political ecology, and disaster risk and resilience studies who were critical of this alarmist position emphasized the complexity and multi-causality of migratory processes and argued the environment cannot be isolated as a lone variable in explaining why someone does or does not migrate (Suhrke, 1994; Black, 2001; Castles, 2002). Research looking beyond push-pull factors have demonstrated that environmental changes interact with other drivers of migration, including political, economic, cultural, and social factors (Lee, 2001; Black et al., 2011; McLeman, 2013). Perspectives from political ecology emphasize how vulnerability to environmental changes and disasters largely flows from economic, social, and historical forces and conditions; the extent to which a community is vulnerable to climate change is then highly contextual (Oliver-Smith, 2012; Stojanov et al., 2014). Intersectional studies of climate vulnerability and migration outcomes demonstrate that gender, class, race and ethnicity, nationality, relation to institutions, and access to migratory networks, among other factors, all mediate one’s ability to respond to environmental stressors, leading to different migration outcomes for individuals exposed to the same environmental conditions (Chindarkar, 2012; Gioli et al., 2014; Djoudi et al., 2016). Movement in response to climate change can also take diverse forms, including displacement, voluntary movement, planned relocation, and immobility (Renaud et al., 2007; Black et al., 2013). To date, empirical research on how environmental factors influence migration to date has suggested that, in the context of climate change, most migration is within nation-state borders, short-term or temporary, from rural to urban areas, while also being contextually dependent and shaped by pre-existing relations of power and inequality (Hoffmann et al., 2020; Kaczan and Orgill-Meyer, 2020).

Such research draws different conclusions from earlier depictions of mass migration caused by climate change, in which climate change is not necessarily the sole cause of mobility, but rather climate change interacts with numerous other factors that influence migratory decisions and a linear relationship between environmental changes and cross-border migration is not a given. Importantly, research has also critically investigated this ‘rejection of determinism’ and ‘move to complexity’ that has now become hegemonic in climate migration studies, stressing that reducing climate change and migration to mere ‘complexity’ can potentially underestimate or ignore structures of power that have both facilitated the climate crisis and its uneven outcomes as well as regimes of mobility, such as colonial and racist histories (Baldwin, 2022).

Researchers have considered knowledge production of climate-related migration and how these can further reproduce stigmatization, as research priorities from governments and other hegemonic institutions remain focused on predicting future climate migration in the interest of border security and migration management (Boas et al., 2019). Additionally, climate migration in international policymaking circles is continually presented as a phenomenon that can be managed and prevented through policy informed by research (Durand-Delacre et al., 2021). Since the early stages of research into climate change and migration, it has been emphasized by researchers that it is incredibly difficult to empirically distinguish between climate and non-climate migrants, and climate migrants are not a migration category of their own (Black et al., 2011; Mayer, 2013; Nicholson, 2014; Bates-Eamer, 2019). Despite this, researchers and policymakers continue to speak about climate migration as a defined phenomenon, even if they accept this complexity and ambiguities in definition. As argued by Nash (2018), this conceptual paradox leads to a ‘self-perpetuating’ cycle of research, in which climate migration is identified by both researchers and political actors as a problem that needs to be investigated, and a lack of conceptual clarity around climate migration leads to the conclusion that more research needs to be done to understand the phenomenon more thoroughly. It has also been argued by researchers that while ‘climate migration’ is difficult to delineate empirically, it is a discursive reality and a narrative that connects a series of phenomena into an issue that can be researched and governed (Bettini, 2013b, 2014). It has also been shown that there is an ‘uneven geography’ of climate migration research, in which certain regions of the world are intensely investigated by climate migration researchers while other areas are not, reifying pre-existing assumptions that certain areas in the Global South are climate migration ‘hot spots’ and a security risk for the Global North (Piguet et al., 2018). Such insights highlight how hegemonic framings of climate migration are linked to state-centered perspectives of migration, as future human movement in the context of climate change is seen as a problem that needs to be controlled by nation-states.

## 4 The climate mobilities paradigm

Critical climate migration researchers have questioned the hegemonic framing of climate-induced migration as a looming global catastrophe by showing how quantitative projections of mass climate migration are methodologically flawed and anchored in environmental determinism (Gemenne, 2011; Nicholson, 2014), emphasizing how such narratives are a tool for neoliberal governance and justify increased border securitization and restrictive migration policies (Bettini, 2013a, 2014; Felli, 2013; Boas et al., 2019), demonstrating how such narratives are rooted in colonial power structures and have racializing effects (Baldwin, 2013, 2022), and calling for new framings on how researchers, policymakers, and the public conceive and frame climate mobility (Durand-Delacre et al., 2021; Boas et al., 2022). Critical climate migration researchers have turned to other framings of mobility and movement, often using the terminology of de-exceptionalizing movement and the mobilities framework, to help understand climate-related mobilities that challenge state-centered understandings of migration.

Some of the first arguments to de-exceptionalize climate-relataed mobility arose out of researchers critiquing the securitization of climate-induced migration, arguing that discourses of climate-induced migration were leading to the creation of a new ‘state of exception’, and it is imperative to not reify the trope that mass climate-induced migration amounts to crisis (Biermann and Boas, 2010; Hartmann, 2010; Bettini, 2012). Researchers from the fields of human geography also added that humans have always moved in response to a variety of factors, including environmental changes, and in this sense climate-related mobility is not exceptional but rather foundational (Baldwin et al., 2019). Such critiques led to the development of the *climate mobilities* concept, which promotes a shift ‘from migration to mobility’ (Nature Climate Change, 2019), rooted in concerns with how the term ‘climate migration’ has come to signify mass, international migrations with little empirical evidence (Boas et al., 2019). The climate mobilities perspective adopts the new mobilities paradigm (Sheller and Urry, 2006) which challenges the fixedness of territories and the view that sedentarist lifestyles are the norm, as well as focusing on how the movement of people and things are shaped by interrelated and unequal power relations (Sheller, 2018). Climate mobilities research then aims for more nuanced analyses of the relationship between climate change and migration, including the understanding that climate mobilities is ‘not necessarily novel and exceptional, but as deeply embedded within historical, current and evolving practices as mobility’ (Boas et al., 2022, p. 3368). Climate mobilities also sees climate change as being relational and highly contextual rather than being the primary cause for mobility, and also pays attention to the subjective experiences of those affected by the climate change-mobility nexus and ‘mobility regimes’ that shape the possibilities of movement (Wiegel et al., 2019). The climate mobilities perspective also draws on Mimi Sheller’s (2018) concept of mobility justice, in which both climate-related risk and migrant precarity are conceptualized as being caused by deep histories of uneven mobilities, reinforced by capitalism, colonialism, and fossil fuel-related extraction.

In recent years, those in the climate mobilities field have also considered how insights from critical migration studies, particularly literature on borders, bordering, and the ‘demigranticization’ of research on migration and integration (Dahinden, 2016), can enrich climate mobilities research (see Boas et al., 2024). This includes questions such as how borders and bordering processes can problematize certain climate mobilities, particularly those in the Global South, as well as how assumptions in policymaking and governance that some areas are safe and some places are lost to a changing climate. Further, such inquiries also call on climate mobilities researchers become more reflexive on how ‘our own frames of analysis enact bordering processes and play into political and public debates about who or what is worth saving’ (Boas et al., 2024, p. 523).

In addition to focusing on bordering, methodological de-nationalism can be a useful approach for those studying climate mobilities to attend to the multitude of ways people may be mobile or immobile in climate change contexts. Manifestations of climate-related movement are present across the globe, both in the Global South and Global North, ranging from abrupt short-term displacement to planned relocations to slow-onset changes interacting with numerous other factors affecting one’s decision to move, often within nation-state borders (Hoffmann et al., 2020; Kaczan and Orgill-Meyer, 2020). Researchers have noted the inequalities embedded in terms such as climate refugee or climate migrant; those in the Global South are labelled in dominant discourses as future climate refugees/migrants who will inevitably have to move, whereas those in the Global North exposed to climate risks are often not considered to be climate migrants (Piguet et al., 2018; Hiraide, 2023). Research has also shown how global and regional power structures impact the outcomes of those affected by ecological disasters and climate change within states, and marginalized populations who are formally considered citizens are often placed at risk and neglect by the governments supposed to protect them (Marino, 2012; Khoshneviss, 2024). Methodological de-nationalism can therefore allow researchers to go beyond solely focusing on international climate migration to see how such cases are linked to more local contexts through shared experiences. Further, turning attention to ‘climate mobility regimes’ rather than ‘climate migration’ can bring attention to how distinctions between citizens and migrants, bordering practices, racial logics, and national belonging are being reinforced and reconstructed by nation-states in response to new challenges brought forth by climate change.

## 5 Decolonial perspectives in climate change and mobility

Research on Indigenous mobility traditions have also added nuance and critique to commonly held assumptions about climate change and migration, as well as challenging notions that migration is exceptional. According to Ramirez (2020), studies of migration that assume migration began with the rise of the nation-state overlook histories and understandings of mobility that pre-dated the nation-state order, including Indigenous traditions of mobility. Whyte et al. (2019) outline how Indigenous philosophies, such as Anishinaabe, have conceptions of movement and mobility that view mobility, change, and fluidity as integral for resilient societies and resettlement as a constant reality. Perspectives from Oceania also show alternative conceptions of borders and mobility. As explained by Hau’ofa (1994), contrasting to the colonial, nation-state centered view that Pacific Islands are ‘small,’ isolated, and therefore dependent on more powerful nations due to their physical land mass and position on the periphery of the global economy, Indigenous perspectives view the Pacific as a ‘sea of islands,’ including not just land but the sea and sky, with a rich history and cosmology. Further, seafaring and movement between islands was vital for the flourishment of cultural, social, and political life (Hau’ofa, 1994; Diaz, 2011). Such insights complicate views of the nation-state and sedentarism being natural features of the social order. In the context of climate change and mobility in the Pacific, Yates et al. (2023) argue that instead of portraying those from i-Kiribati people who are compelled to move due to climate change as victims, reconceptualizing climate mobilities from the perspectives of Pacific navigation traditions maintain both dignity and cultural preservation for those who move despite the indignities and limitations of neoliberal migration frameworks.

Insights from research on settler colonialism and internal bordering have also shown how ongoing colonial and capitalist power structures have sought to limit and curtail Indigenous mobilities, resulting in anti-adaptive conditions and climate injustice (Coleborne, 2015; Marino, 2015; Whyte et al., 2019). In her fieldwork in Shishmaref, Alaska, Marino (2012, 2015) illustrates how the community’s immobility is the outcome of colonial strategies that sought to Christianize, educate, and render sedentary Indigenous peoples in the Arctic, while simultaneously facilitating resource extractive industries, such as fishing and oil. Centering issues of colonialism, capitalism, and including Indigenous intellectual traditions in discussions about mobility and climate change adds important nuance and critique to hegemonic conceptions of mobility and climate change which isolate climate change as the causal factor of migration and resettlement (Baldwin et al., 2019; Whyte et al., 2019), offering a more holistic approach. Research in this field has also challenged the supposed inevitability of climate migration that is often assumed in policymaking contexts. In her fieldwork on the island of Funafala, Farbotko (2022) illustrates how community members emphasize habitability in contrast to international governance perspectives that label their island as doomed to be lost to climate change; such insights highlight the inequalities in adaptation projects raise the issue of communities being able to adapt to climate change on their own terms.

Whyte (2021) uses the term epistemologies of crisis to describe the understanding of certain phenomena as new, arguing that climate change narratives often employ a crisis epistemology. According to Whyte, crisis epistemologies are based around a presentist unfolding of time, in which it is understood that such crises are both unprecedented and urgent; therefore, they must be responded to quickly using solutions-oriented approaches. However, such presentist conception of climate change make it possible to willfully obscure underlying structural causes and mask numerous forms of power, including colonialism and global capitalism. Further, the overarching sense of urgency makes it possible to suspend or ignore questions of ethics or justice in the name of finding and implementing climate ‘solutions.’ Illustrative of this is how numerous ‘clean’ energy projects around the globe are unjust and harmful to Indigenous peoples, including continuing processes of dispossession, economic deprivation, and land desecration (Sehlin MacNeil, 2017; Dorn, 2022; Össbo, 2022; Zografos, 2022). Discourses on climate migration can be said to also employ a crisis epistemology, as it is often assumed that climate migration is an unprecedented phenomenon that is in dire need of solutions and management, obscuring structural factors and relations of power. Contrastingly, Whyte (2021) identifies alternative ways of understanding social formations that he labels as epistemologies of coordination, which emphasize relationality, moral bonds, and mutual responsibilities. In the context of climate-induced migration, research and policies based around epistemologies of collaboration, that seek to find more just solutions and are centered around questions of responsibility and reciprocity, could lead to more sophisticated and creative solutions to pressing environmental problems, rather than continued practices of neoliberal migration management (Whyte et al., 2019).

## 6 Climate mobilities, migration management, and reproducing inequalities

There is growing research on the experiences of people who migrate internationally at least partly due to climate change, with much research coming from Oceania. Such research highlights how state-centric approaches to migration, centered around migration management and the deterrence of ‘undesirable’ migrants establishing residency within a nation-state, reproduce further inequality and precarity in the context of climate change.

In dominant climate migration narratives, residents from Oceania are often portrayed as being as being on the front lines of climate change, as their homelands are jeopardized due to rising sea levels. Research on the lived experiences and perspectives of those living in island states show variegated perspectives, including rejecting the label of ‘climate migrant’ and instead calling on the international community to reduce emissions (McNamara and Gibson, 2009; Farbotko and Lazrus, 2012), a preference for in-situ adaptation measures and remaining in their homelands (Perumal, 2018; Farbotko, 2022), and emphasizing concerns over political, territorial, and cultural rights (Walshe and Stancioff, 2018). However, environmental deterioration has impacted Pacific Islands communities, and with limited adaptation options as a result of colonization and extraction in the Pacific (Bordner et al., 2020), many Pacific Island governments and residents are considering alternative mobility solutions (Thornton et al., 2020). In 2014, the former President of Kiribati introduced the ‘migration with dignity’ framework as a possible adaptation response to climate change, with the ambitions that residents would be able to choose where, when, and how they moved, alongside the expectation that they would maintain or improve their living standards abroad (Tong, 2014). However, with limited support from host nations the program had to facilitate migration within existing migration frameworks (Kupferberg, 2021; McMichael et al., 2021).

In the case of people who are forced or compelled to migrate due to environmental disasters and climate changes, there is limited policy infrastructure and a human rights protections gap (Biermann and Boas, 2010; Jolly and Ahmad, 2015; McAdam, 2020). While terms such as environmental refugee or climate refugee are commonly circulated by media outlets, activists, and non-governmental organizations, politicians and scholars, such terms do not have a legal basis (Hiraide, 2023). Outside of asylum and refugee law, national governments, which tend to categorize migration into a binary of forced or voluntary, do not see environmental changes as a possible reason for migration (Bates-Eamer, 2019). Research has shown that when people affected by climate change pass through migration management regimes, they often experience misrecognition and are put in a state of legal limbo due to asylum and migration policies; resultingly, they are often compelled to enter various labour migration schemes, often temporary in nature, or migrate through other channels, including living undocumented (Bates-Eamer, 2019; Stanley, 2021; Yates et al., 2023). Yates et al. (2023) demonstrate how this.

As noted by researchers, as those who migrate due to climate change do not have protection as refugees and migration systems often do not consider environmental factors as a possible reason for migration, they are often compelled to enter various labor migration schemes, often short-term in nature, or migrate through other channels, including living undocumented (Skillington, 2015; Bates-Eamer, 2019; Nguyen and Kenkel, 2021; Yates et al., 2023). Yates et al. (2023) demonstrate how this state of legal limbo and nonrecognition, as well as neoliberal migration systems, limited the options of climate migrants from i-Kiribati and Tuvalu to Aotorea New Zealand. While some participants migrated on employment-dependent migration schemes, others entered on short-term visas and stayed as irregular migrants. As explained by the authors, participants became trapped in a purgatorial state of ‘deportability’, impacted their ability to make a life in New Zealand and making them vulnerable to exploitation. According to Nguyen and Kenkel (2021), once those who migrated to New Zealand from Tuvalu became irregular, it became considerably harder to both gain regularized status and find jobs outside of low-wage, informal employment. In this sense, the denial of haven from climatic breakdown can be seen as amounting to a form of administrative violence and climate injustice against those who are severely impacted by climate change (Yates et al., 2023).

Such insights highlight what is at stake when discussions and research around climate change and migration remain uncritically centred around border security, controlling migration, and upholding nation-state sovereignty. Through the categorization of migrants, bordering and externalization practices, and migration policies that create temporariness and restrict inclusion into the state, contemporary border and migration policies prioritize nation-state sovereignty and economic interests over the protection of people on the move (De Genova et al., 2014; Oelgemoller, 2017; Bates-Eamer, 2019; Moreno-Lax and Lemberg-Pedersen, 2019). In this sense, calls for rights-based frameworks for climate migrants/refugees that naturalize nation-state migration regimes, instead of interrogating the indignities produced by these structures, will do little to protect those affected by climate change. New approaches to understanding climate mobilities, such as theoretical orientations that de-centre the nation-state and de-exceptionalize migration, as well as decolonial perspectives that attend to multiple histories and understandings of movement that also attend to underlying causes such as capitalism and colonialism, can lead to the creation of more holistic and innovative climate solutions.

## 7 Discussion

In discussions of climate change and mobility in research and policymaking contexts, there are often calls for ‘more and better’ data, and that climate migration needs to be better understood by both researchers and political actors in the interest of creating policy and responses to climate migration. However, the drive for more robust data can have a counterproductive effect of sidelining already well-established knowledge in climate migration research, calling for an interrogation of what kind of questions are being asked and what knowledges are being produced when it comes to climate change and mobility, and what are the outcomes and imperatives of these knowledges (Nash, 2018; Durand-Delacre, 2022).

This article has argued that mainstream knowledge production of climate-related mobility upholds state-centric approaches to migration and migration management. This includes the continued assumption that climate change will result in mass cross-border migration, that climate-related migration is an issue of national security, and that future climate migration should be prevented and contained. Such assumptions center views of migration that conceptualize human mobility as a deviation from the norm or a problem for governments, which, echoing Bridgit Anderson (2019) carries both epistemological and ethical challenges for both researchers and other actors. Further, existing migration policy frameworks and migration management practices continue to reproduce inequalities for those who are in the nexus of climate change and migration through unequal access to mobility. While most discussions on justice and equity for present and future climate migrants emphasize legal recognition as a pathway for justice, legal recognition alone will do little for those affected by climate change if current migration management practices remain unchanged. In this sense, a critical climate justice (Sultana, 2022) needs to be conceptualized alongside migrant justice, as articulated in Sheller’s (2018) mobility justice framework.

This article has brought together different theoretical dimensions in both critical migration studies, such as the new mobilities paradigm, methodological de-nationalism, and decolonial perspectives in migration studies, into conversation with new approaches in climate mobilities research. Such perspectives provide more holistic conceptions of human movement, as well as historicizing and contextualizing ‘new’ phenomena as continuations of already existing relations of power and inequality. Importantly, such insights can also lead to more innovative approaches to environmental change and mobility, rather than the continuation of neoliberal mobility regimes. While this article has aimed to bring these perspectives together in a way that is more exploratory, important work remains to be done in regards to investigating how can perspectives that de-centre nation-states and attend to diverse histories and understandings of mobility be fully realized in a world that continues to build walls and borders. In response to Nash’s (2018) call to interrogate what is ‘thinkable’ in the context of climate change and mobility, more holistic and decolonial perspectives on migration and movement can shift our analytical focus to structural conditions underscoring climate change and migration and the vulnerabilities, rights, and freedoms of people, rather than narratives of crisis and control.
